# Molecular Recognition of the Neurotransmitter Acetylcholine by an Acetylcholine Binding Protein Reveals Determinants of Binding to Nicotinic Acetylcholine Receptors

**DOI:** 10.1371/journal.pone.0091232

**Published:** 2014-03-17

**Authors:** Jeppe A. Olsen, Thomas Balle, Michael Gajhede, Philip K. Ahring, Jette S. Kastrup

**Affiliations:** 1 Department of Drug Design and Pharmacology, Faculty of Health and Medical Sciences, University of Copenhagen, Copenhagen, Denmark; 2 NeuroSearch A/S, Ballerup, Denmark; 3 Faculty of Pharmacy, The University of Sydney, Sydney, New South Wales, Australia; 4 Aniona ApS, Ballerup, Denmark; H. Lee Moffitt Cancer Center & Research Institute, United States of America

## Abstract

Despite extensive studies on nicotinic acetylcholine receptors (nAChRs) and homologues, details of acetylcholine binding are not completely resolved. Here, we report the crystal structure of acetylcholine bound to the receptor homologue acetylcholine binding protein from *Lymnaea stagnalis*. This is the first structure of acetylcholine in a binding pocket containing all five aromatic residues conserved in all mammalian nAChRs. The ligand-protein interactions are characterized by contacts to the aromatic box formed primarily by residues on the principal side of the intersubunit binding interface (residues Tyr89, Trp143 and Tyr185). Besides these interactions on the principal side, we observe a cation-π interaction between acetylcholine and Trp53 on the complementary side and a water-mediated hydrogen bond from acetylcholine to backbone atoms of Leu102 and Met114, both of importance for anchoring acetylcholine to the complementary side. To further study the role of Trp53, we mutated the corresponding tryptophan in the two different acetylcholine-binding interfaces of the widespread α4β2 nAChR, *i.e.* the interfaces α4(+)β2(−) and α4(+)α4(−). Mutation to alanine (W82A on the β2 subunit or W88A on the α4 subunit) significantly altered the response to acetylcholine measured by oocyte voltage-clamp electrophysiology in both interfaces. This shows that the conserved tryptophan residue is important for the effects of ACh at α4β2 nAChRs, as also indicated by the crystal structure. The results add important details to the understanding of how this neurotransmitter exerts its action and improves the foundation for rational drug design targeting these receptors.

## Introduction

Acetylcholine (ACh) is an important neurotransmitter in the central and peripheral nervous system where it mediates fast excitatory neurotransmission by binding to nicotinic acetylcholine receptors (nAChRs) [1]. Significant progress in understanding this process has been made recently by structural studies of receptors and homologues proteins [2].

Insights into the organization of the full-length nAChRs have been achieved by electron microscopy of nAChRs from *Torpedo marmorata* [3], [4]. Also, X-ray crystallography has been successfully undertaken on homologues ion channels, including a proton activated channel from the bacterium *Gloeobacter violaceus* (GLIC) [5], [6], an amino acid activated channel from the bacterium *Erwinia chrysanthemi* (ELIC) [7], [8] and a glutamate activated chloride channel from the invertebrate *Caenorhabditis elegans* (GluCl) [9]. From these structures, it is even possible to form reasoned hypotheses about the overall movements that occur in response to neurotransmitter binding [4], [5], [6]. Detailed structural information on the extracellular part of nAChRs has been achieved by the 1.9 Å resolution structure of the extracellular domain of the mouse α1 subunit [10]. This structure provides information on the subunit structure but, as it is crystallized as a monomer, it does not contain an intact ligand-binding site. A nAChR ligand-binding domain homologue, the ACh binding protein (AChBP), has proven a uniquely well-suited system for the study of receptor-ligand interactions [2], [11]. AChBP forms a pentameric structure where the interfaces between the subunits provide an ACh binding pocket that is homologous to the binding pocket of nAChRs [11], [12].

To date, more than 50 structures of agonists and antagonists bound to AChBP have been deposited in the PDB [13], [14], including a structure of AChBP with the ACh analog carbamoylcholine [15].

AChBPs have been isolated from different snail species such as *Aplysia californica* (*Ac*-AChBP) and *Lymnaea stagnalis* (*Ls*-AChBP). While these proteins resemble each other in many respects, important differences are noted. *Ac*-AChBP has so far been the most widely used for crystallization experiments; however, in the context of nAChRs the binding pocket of *Ls*-AChBP shows better resemblance as it contains all the five aromatic residues fully conserved in nAChRs. Further, despite a few amino acid differences near the binding site, correlation of binding affinities from *Ls*-AChBP to α7 [16] and α4β2 [17] nAChRs has been demonstrated for different compound series. This suggests that *Ls*-AChBP can successfully be used to study details of binding to nAChRs. To improve resemblance to nAChRs, AChBP has been mutated in both comprehensive [18] and strategic approaches [19]. Recently, an *Ac*-AChBP mutant was crystallized with two copies of the endogenous neurotransmitter ACh binding at two sites in the same pocket [20]. This *Ac*-AChBP mutant has a cysteine residue in place of a highly conserved tryptophan residue at the binding site interface of nAChRs (Trp82 in the β2 subunit) that is known to form part of the ACh binding pocket [21]. This leaves unanswered questions about the details of ACh binding to intact AChBPs and nAChRs. An example of the importance of such details of binding is the observation that a few residue differences at the interfaces in the heteromeric (α4)_3_(β2)_2_ nAChRs were shown to have important implications for the effect of ACh binding [22]. Thus, better understanding of the binding characteristics of ACh in nAChRs is clearly important when designing new drugs specifically targeting individual sites.

**Figure 1:**
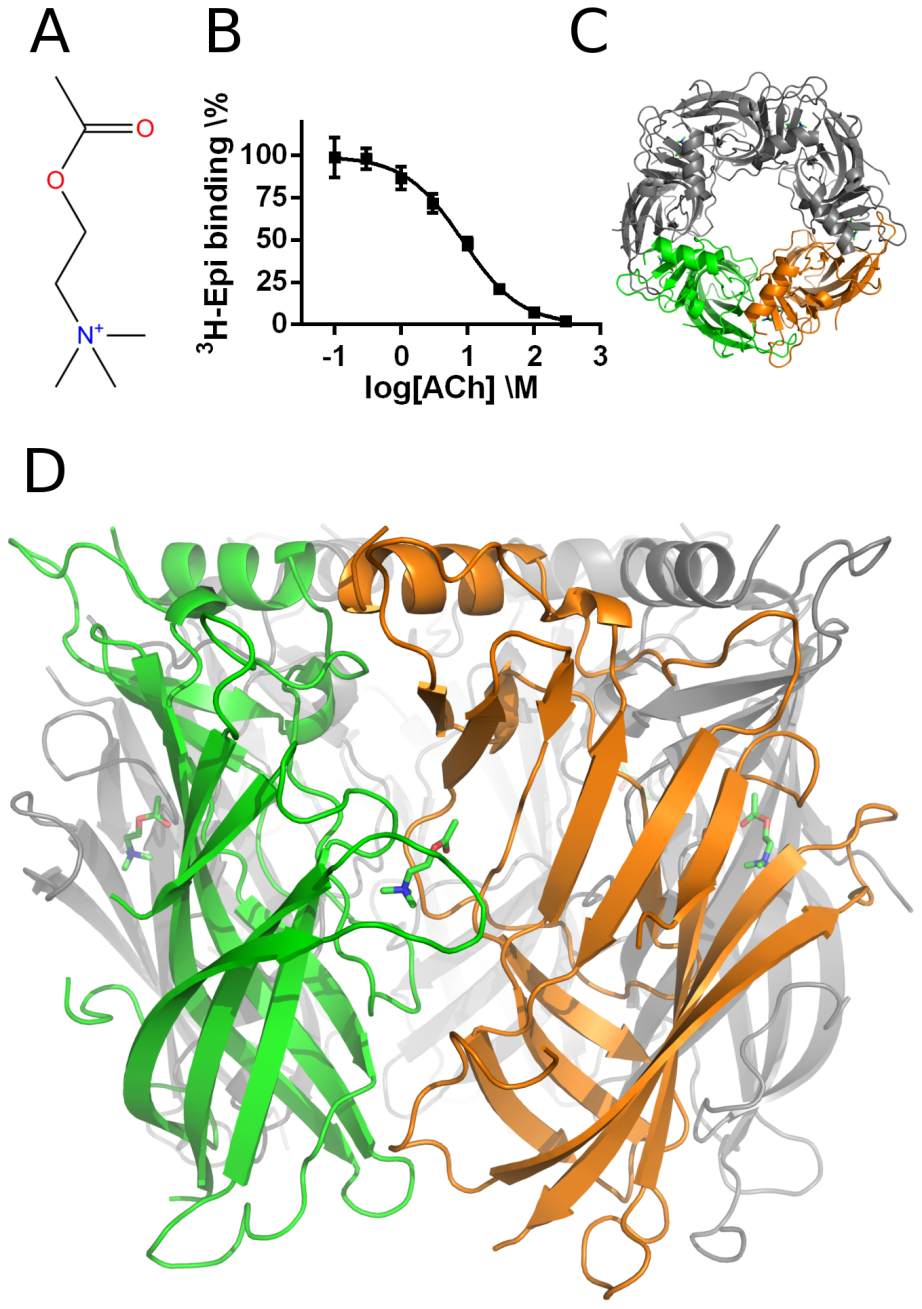
Structure of ACh bound to *Ls*-AChBP. (**A**) The structure of acetylcholine (ACh). (**B**) Displacement of tritium-labeled epibatidine (^3^H-Epi) bound to *Ls*-AChBP by ACh was used to determine the IC_50_ value of ACh. The data points shown are from one determination of the IC_50_ value. The average of three such experiments were converted to the K_i_ value of ACh by the Cheng-Prusoff equation. (**C**) Top-view of a cartoon representation of the structure of one *Ls*-AChBP pentamer with an ACh molecule bound in each interface. (**D**) Side-view of a cartoon representation of the *Ls*-AChBP with ACh shown in green stick representation. The ACh molecule is located between two colored subunits: the green subunit forms the principal side of the binding pocket, (+) interface, while the orange subunit forms the complementary side, (−) interface.

Here, we report the crystal structure of ACh bound to *Ls*-AChBP (Figure 1A). This study is the first to provide a crystal structure of ACh in a binding pocket containing all five aromatic residues conserved in all mammalian nAChRs. As we observe cation-π interactions between ACh and Trp53 on the complementary side of the intersubunit binding interface of *Ls*-AChBP, functional studies on α4β2 nAChRs were performed using oocyte voltage-clamp electrophysiology. Mutation of the corresponding tryptophan affected ACh-mediated gating, underscoring the functional importance of this residue on the complementary side of the binding interface. Hence, this study provides further insight into the molecular basis of ACh action in nAChRs.

## Results and Discussion

To ensure that ACh binds with an affinity at *Ls*-AChBP comparable to that at nAChRs, we determined the binding affinity of ACh to a construct of *Ls*-AChBP linked to a 5HT3 receptor ion channel. We have previously evaluated this construct for use in a [^3^H]-epibatidine binding assay [17]. The K_i_ value of 1.5±0.38 μM (Figure 1B; n = 3) is in good agreement with the K_d_ determined by fluorescence spectroscopy [16], and by ITC [15], which also shows that the affinity is about a factor ten less than for carbamoylcholine. Therefore, the affinity is comparable to the relatively low affinity seen for ACh binding at α7 nAChRs [15]. Although ACh affinity at α4β2 receptors is 50-fold higher, the relative affinities for a series of compounds at these receptors were reproduced at *Ls*-AChBP [17], implicating that it is a useful model system for this receptor subtype as well.

### Structure of ACh bound to *Ls*-AChBP

**Figure 2:**
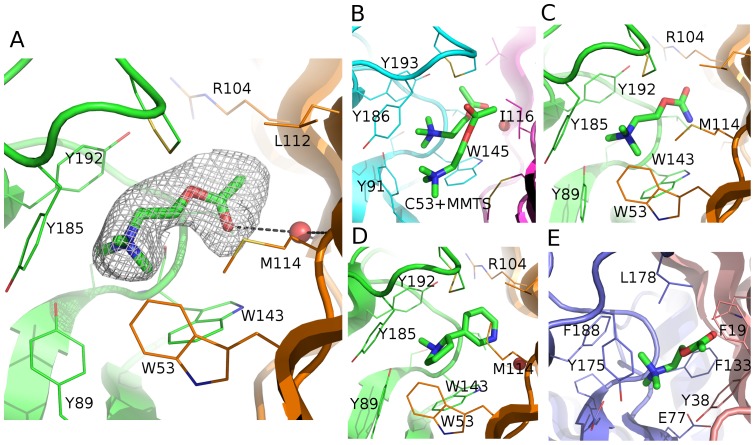
Close-up view on the ACh-binding site and comparison to other structures. Structures are shown with ligands as green sticks and side chains within 5 Å of ligands as lines. (**A**) ACh bound to *Ls*-AChBP, as reported here, with principal side-chain carbon atoms in green and complementary side-chain carbon atoms in orange. The mesh around ACh corresponds to a 2mFo-DFc omit map calculated in PHENIX, shown at a level of 1ó and carved at 2.2 Å around the ligand. (**B**) ACh bound to a MMTS-modified Y53C mutant of *Ac*-AChBP [20], with principal side residues in cyan and complementary side residues in magenta. (**C**) Carbamoylcholine bound to *Ls*-AChBP [15] with coloring as in (A). (**D**) Nicotine bound to *Ls*-AChBP [15] with coloring as in (A). (**E**) ACh bound to ELIC [24], with principal side residues in blue and complementary side residues in salmon.

*Ls*-AChBP was crystallized in complex with ACh in space group C2 by the vapor diffusion method. A complete data set was collected to 2.6 Å resolution and the asymmetric unit of the crystal contains two pentamers (Figure 1), corresponding to a total of 2024 residues built. For statistics on data collection and structure refinement, see Table 1. Additional electron density for a single ACh molecule was present at all ten subunit interfaces (Figure 2A). The ligand adopts a similar location and conformation (average RMSD of 0.04 Å between all ACh non-hydrogen atoms) at each interface and binds under a closed loop C (Figure 2A and Figure S1). Additional F_o_–F_c_ electron density in the vicinity of the ester methyl group of ACh is observed in some interfaces, indicating flexibility in the position or conformation of the ligand ester group (Figure S2). The C loops have a uniform degree of closure across the interface (∼7.7 Å) when measured from the disulphide bridge (C atom of Cys187) to the backbone carbonyl oxygen atom of Trp143. This is comparable to the degree of closure that we have previously observed for partial α4β2 nAChR agonists [17], [23].

**Table 1:** Data collection and refinement statistics.

Data collection	
Space group	C2
a, b, c (Å)	236.85, 73.15, 132.47
α, β, γ (°)	90.0, 101.5, 90.0
Mosaicity (°)	0.56
Pentamers/AU^a^	2
Resolution range (Å)	48.29–2.60 (2.74–2.60)^b^
Reflections, obs.	222848
Reflections, unique	68170
Multiplicity	3.3 (3.2)
Completeness (%)	99.2 (99.9)
R_merge_ (%)^c^	10.1 (29.6)
I/σI	5.0 (2.0)
Wilson B (Å^2^)	35
Refinement	
No. of residues:	
Protein	2024
ACh	10
PEG400	12
Water	408
R(work) (%)^d^	17.9 (22.8)
R(free) (%)^d^	23.9 (34.0)
RMSD, bonds (Å)	0.009
RMSD, angles (°)	1.4
Ramach. outliers (%)^e^	0.1
Ramach. favored (%)^e^	98.8
Average B (Å^2^):	
Protein	35
ACh	27
Water	31
PEG400	50

a AU: asymmetric unit of the crystal.

b Numbers in parenthesis correspond to the outer resolution bin.

c A measure of agreement among multiple measurements of the same reflections. R_merge_ is calculated as follows: I_i_(hkl) is the intensity of an individual measurement of the reflection with Miller indices hkl, and I(hkl) is the intensity from multiple observations:R_merge_ = ∑_hkl_∑_i_|I_i_(hkl)−I(hkl)|/∑_hkl_∑_i_|I_i_(hkl)|.

d R(work) = ∑_hkl_| F_obs_−F_calc_ |/∑_hkl_|F_obs_|, where F_obs_ and F_calc_ are the observed and calculated structure factor amplitudes, respectively. The free R-factor, R(free), is computed in the same manner as R(work), but using only a small set (5%) of randomly chosen reflections not used in the refinement of the model.

e The Ramachandran plot was calculated using PHENIX.

ACh adopts a fully extended conformation in *Ls*-AChBP (average torsion angles with the range in parenthesis: T_CCOC_ = −178° (−179−(−178)°), T_COCC_ = −145° (−150−(−143)°), T_OCCN_ = 169° (168−173°) and T_CCNC_ = −153° (−154−(−151)°)). This conformation of ACh is different from the conformation observed in the structure where it is co-crystallized with *Ac*-AChBP (Figure 2B), in which the tryptophan on the complementary side is not present. In particular, it seems possible that the quaternary ammonium group has adjusted its orientation to accommodate interactions to both Trp53 and Trp143. The observed conformation is more similar to that of carbamoylcholine in *Ls*-AChBP (Figure 2C), but the carbonyl oxygen atom of ACh is pointing in the opposite and energetically more favorable direction compared with the carbonyl oxygen of carbamoylcholine [15]. The conformation of ACh can be assigned based on an interaction to a binding-site water molecule, which is clearly visible in the density in the ACh structure, but was not modeled into in the carbamoylcholine structure. This water molecule was also seen in both the *Ls*-AChBP structure with nicotine [15] (Figure 2D), and in the *Ac*-AChBP structure with ACh (Figure 2B). A different conformation of ACh is seen in the structure of ELIC [24], where T_OCCN_ corresponds to a *gauche*(+) conformation (Figure 2E).

In both the *Ls*-AChBP and ELIC structures, ACh binds within an aromatic box. However, the residues comprising the aromatic box and the specific contacts possible differ between the two proteins. In particular, Trp53 and Trp143, that both are important for agonist action of ACh, are not found in ELIC. It is therefore interesting to note that ACh acts as an antagonist at ELIC [24].

### Interactions between *Ls-*AChBP and Ach

Since the AChBP:ACh complex was crystallized as two pentamers with a total of ten ligands present, it allows us to make repeated observations of interaction distances. The resulting average is taken as the best way to represent the distances and will be used in the following with the range in parenthesis. The ligand-protein interactions are characterized by contacts to an aromatic box formed primarily by residues on the principal side of the interface (residues Tyr89, Trp143, Tyr185 and Tyr192; Figure 2A and Figure S1) but also by Trp53 on the complementary side. At the principal side, a pocket is formed well adapted to accommodate ACh, where the ligand is wedged in between the Cys187–Cys188 disulphide bridge and Trp143. The backbone carbonyl oxygen atom of Trp143 interacts with the carbon adjacent to the ammonium group of ACh at a distance of 3.1 Å (3.0–3.2 Å), similar to a previous observation for carbamoylcholine [15]. Trp143 and one of the methyl groups of the trimethylammonium group of ACh are positioned 3.6 Å (3.4–3.7 Å) from each other, when measured from the centroid of the benzene part of the indole in the tryptophan side chain. This geometry is consistent with a cation-π interaction that has also been observed in unnatural amino acid mutagenesis studies [25]. At the complementary side of the interface, the pocket is less tight around ACh and the contacts more sparse. The anchoring to this side is characterized by a binding site water molecule located 3.5 Å (3.3–3.6 Å) from the carbonyl oxygen atom of ACh. The water mediates hydrogen bonds to the backbone carbonyl oxygen atom of Leu102 and the backbone nitrogen atom of Met114 (Figure 2A). This water molecule was not found in the structure of *Ls-*AChBP with carbamoylcholine [15], possibly because it was not resolvable from the density, but is clearly visible as an important interaction partner in the present structure. Furthermore, unnatural amino acid mutagenesis has confirmed the conserved leucine residue to be functionally important [26]. In addition to the water-mediated hydrogen bonds, Trp53 is positioned to interact with the trimethylammonium group of ACh with a distance of 4.9 Å (distance between trimethylammonium nitrogen of ACh and the centroid of the benzene part of the indole in the tryptophan side chain: 5.8 Å (5.4–6.1 Å)), *i.e*. within the 6 Å that has been used as a rough limit when identifying cation-π interactions [27], [28].

### Trp53 adopts two distinct conformations in *Ls*-AChBP

**Figure 3:**
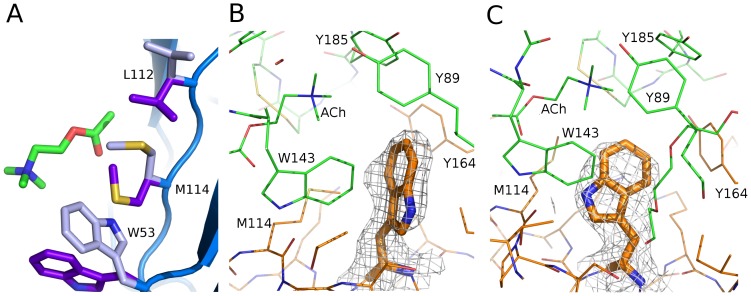
Two conformations of Trp53, Leu112 and Met114. (**A**) On the complementary side of the interface, three residues near ACh adopt two distinct sets of conformations (shown in purple and light-blue sticks, respectively), at some interfaces occurring separately and at other interfaces with both conformations occurring as shown here. (**B**) **and** (**C**) Trp53 is shown in stick representation in the two different orientations observed, in interfaces where distinct orientations are seen. Principle side carbon atoms are colored green, while complementary side carbon atoms are orange. A mesh is shown in each case, corresponding to a partial omit map shown at 1ó and carved at 2 Å around Trp53. The partial omit map was generated using PHENIX by refining the structure after changing all Trp53 residues to alanine, thus alleviating side-chain orientation bias for this residue. (**B**) In one possible orientation, the Trp53 side-chain nitrogen atom is pointing “away” from Met114 with Trp53 and Trp143 aligned for T-type ππstacking. (**C**) In the other conformation, which is favored when a PEG400 molecule is present nearby, the Trp53 side-chain nitrogen atom is pointing towards Met114 and can form a hydrogen bond to the backbone carbonyl oxygen atom of this residue.

When comparing all 10 ACh binding sites in the two *Ls*-AChBP pentamers, it was apparent that Trp53 at the complementary side of the interface adopts two different side-chain conformations (Figure 3A). In four of the binding sites, the side chain of Trp53 adopts a conformation in which it provides a kind of lid at the bottom of the ACh binding site (Figure 3A, purple and Figure 3B). At four binding sites, a PEG400 molecule from the crystallization buffer is located within 4.5 Å of ACh (Figure S3). Apparently without affecting the binding mode of ACh in a direct manner, when these PEG400 molecules are present Trp53 adopts a conformation pointing deeper into the binding site (Figure 3A, light blue and Figure 3C). This causes a correlated conformational change in the side chain of Met114 followed by a change in the side-chain conformation of Leu112 (Figure 3A), giving rise to two distinct populations of ACh binding sites in the structure. At the remaining two binding sites with no PEG400 bound near ACh we see electron density from both populations, indicating mixed conformations at these interfaces. Therefore, it appears that PEG400 is not the sole cause of the flexibility but it is unclear if the flexibility is also present in nAChRs, where Met114 and Leu112 are not conserved [11], and if it has any functional role.

Comparing the environment of Trp53 in each conformation reveals some interesting differences. In one possible orientation (Figure 3B), the Trp53 side-chain nitrogen atom is pointing away from Met114, and Trp53 and Trp143 are aligned for a T-type π-π stacking. The only other interactions of the Trp53 side chain in this orientation are to ACh and Tyr164 where some degree of π-π stacking appears possible. In the other conformation (Figure 3C), which is favored when a PEG400 molecule is present nearby, the Trp53 nitrogen atom is pointing towards Met114 and can form a hydrogen bond to the backbone carbonyl oxygen atom of this residue. Met114 also forms a hydrogen bond to the side-chain nitrogen atom of Trp143. At the same time, the altered orientation of Trp53 provides a less favorable stacking environment with Trp143 and Tyr164. An obvious effect of the individual side-chain conformations of Trp53 is observed on neither backbone trace nor loop C conformation.

### Mutation of a key tryptophan in α4β2 nAChRs influences ACh-evoked gating efficiency

**Figure 4:**
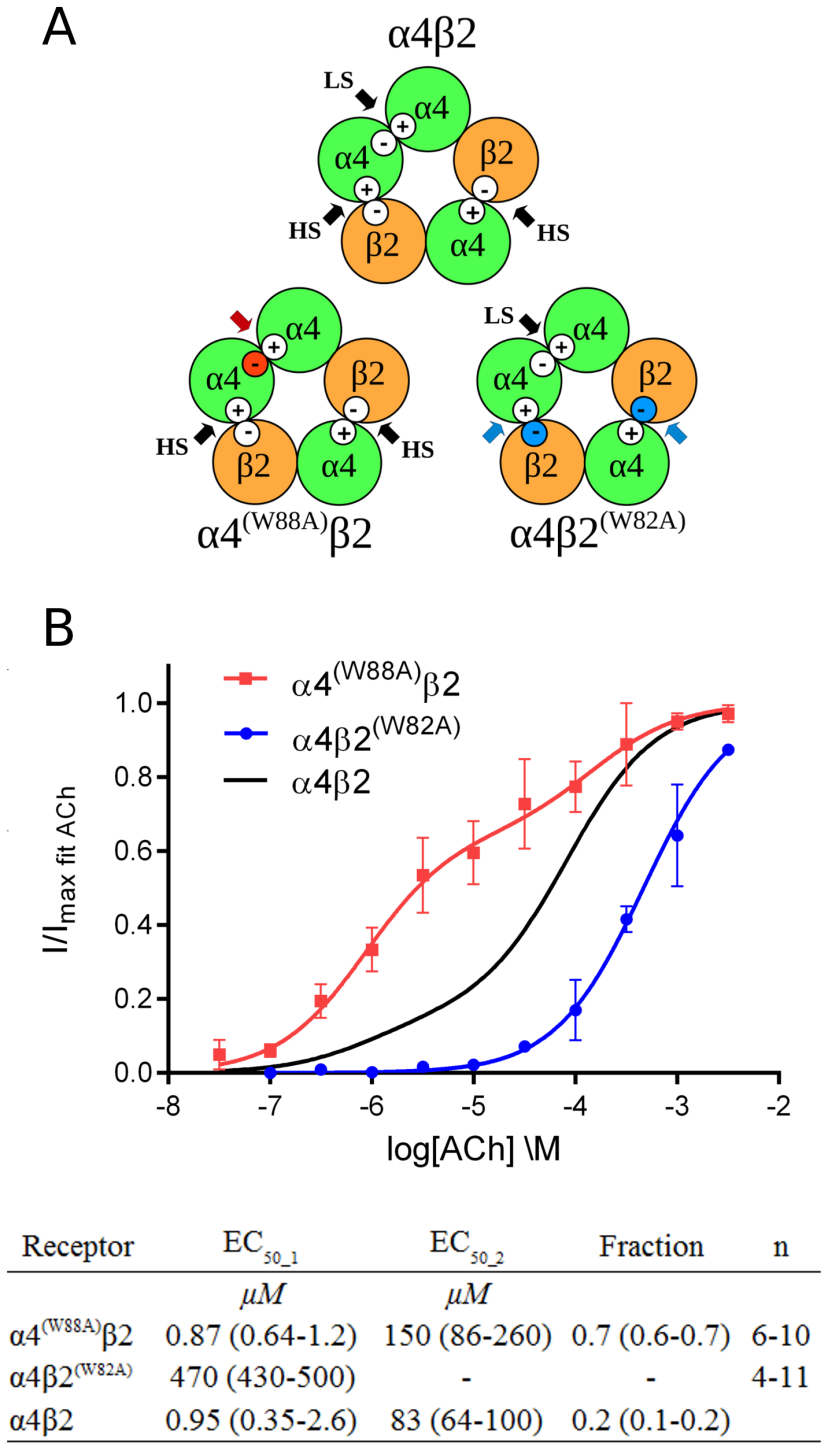
Concentration-response relationships at mutated nAChRs measured by two-electrode voltage clamp on *X. laevis* oocytes. (**A**) α4β2 nAChRs with 3α:2β stoichiometry have three binding sites for ACh (black arrows): two with high sensitivity (HS) and one with low sensitivity (LS). Point-mutation of a central tryptophan residue in the α4 subunit will change the complementary (−) side of the LS site (red circle and arrow). Point-mutation of the corresponding tryptophan in the β2 subunit will change the complementary side of both HS binding sites (blue circles and arrows). (**B**) Concentration-response relationships (CRRs) of ACh at α4^(W88A)^β2 and α4β2^(W82A)^ receptors. The black curve is drawn from previously published data for the ACh CRR at type α4β2 nAChRs with 3α:2β stoichiometry [22], with EC_50_ and fraction values listed below the figure. ‘Fraction’ describes the fraction of the maximum response that is elicited by the high-sensitivity phase. Numbers in parenthesis refer to 95% confidence intervals. ‘n’ is the range of the number of measurements that were made of each point on a curve. An F test was carried out in GraphPad Prism 4 against the null hypothesis of a monophasic fit, which was rejected for α4^(W88A)^β2 (F = 120, DFnN = 2, DFnD = 288) and accepted for α4β2^(W82A)^ (F = 0.73, DFnN = 2, DFnD = 72), where DFnN and DFnD are the degrees of freedom of the numerator and denominator in the F test, respectively.

Compared with previous structures, stronger contacts to the complementary side of the binding interface were observed with the contact from the carbonyl oxygen atom of ACh to a binding site water molecule and cation-π interactions with Trp53. Such strong contacts of ACh are in agreement with the notion that efficacy of an agonist appears to be related to the ability of forming a stable intersubunit bridge [17]. All human neuronal nAChRs has a tryptophan at the position of Trp53 in *Ls*-AChBP. The residues role as a direct ACh interaction partner and the apparent flexibility in the crystal structure suggests that it may have an important functional role. Therefore, we investigated the role of this residue in the 3α:2β stoichiometry of α4β2 nAChRs, where two different binding sites are made from interfaces between α4-β2 or α4-α4 subunits (Figure 4A). Full ACh concentration-response relationships (CRRs) were obtained from two sets of mutated receptors, α4^(W88A)^β2 and α4β2^(W82A)^, using two-electrode voltage-clamp in *Xenopus* oocytes. At α4β2^(W82A)^ receptors, the complementary side tryptophan (corresponding to Trp53 in *Ls*-AChBP) has been exchanged for an alanine in the two α4–β2 binding sites and therefore, the high-sensitivity component of the response was expected to be affected if Trp82 is essential for function. This was indeed the case, and only one EC_50_ value of 470 μM (F_H0 = monophasic fit_ = 0.73, DFnN = 2, DFnD = 72) corresponding to a low-sensitivity component was seen (Figure 4B). At α4^(W88A)^β2 receptors, where only the single α4–α4 binding site has been mutated, the high-sensitivity component would be expected to remain unaltered whereas the low-sensitivity component might change. For this receptor, a biphasic response was seen with EC_50_ values of 0.87 μM and 150 μM. These values are not significantly different from the wildtype EC_50_ values, for either the high-sensitivity (F_H0 = unchanged_ = 0.29, DFnN = 1, DFnD = 286) or low-sensitivity component (F_H0 = unchanged_ = 2.6, DFnN = 1, DFnD = 286). The most noticeable difference at the α4^(W88A)^β2 receptor is instead the marked increase in the fraction of high-sensitivity component from 16% of total current in wildtype [22] to 65% in the mutant receptor. This indicates that ACh is inefficient at contributing to gating through the mutated α4–α4^(W88A)^ interface, thereby leaving most gating as being a consequence of only α4–β2 interface binding. Collectively, marked changes were observed upon mutating the tryptophan corresponding to Trp53 in *Ls*-AChBP in both á4–â2 and á4–á4 interfaces of á4â2 receptors, which suggests that interactions to this tryptophan residue are critical in determining the response to ACh. Since Trp53 appears to be the central contact to the complementary side of the interface, this agrees with the notion that efficacy as an agonist is related to the ability of forming a stable intersubunit bridge [17].

In the crystal structure of AChBP in complex with ACh, the tryptophan corresponding to Trp82 in the β2 subunit and Trp88 in the α4 subunit is positioned for a cation-π interaction with ACh. This interaction was not registered in a previous unnatural amino-acid mutagenesis study [25]; however, the observed functional importance of this tryptophan strongly suggests that it does make an important interaction with ACh, and based on our structure it is likely that an additional cation-π interaction is taking place.

## Conclusions

In conclusion, the structure reported here is the first structure of ACh in a binding pocket containing all five aromatic residues conserved in all mammalian nAChRs. ACh was shown to bind under a closed loop C with a degree of closure comparable to that previously seen for partial α4β2 nAChR agonists. Besides contacts to the aromatic box formed primarily by residues on the principal side of the intersubunit binding interface we observed a cation-π interaction between ACh and Trp53 on the complementary side and a water-mediated hydrogen bond from ACh to backbone atoms of Leu102 and Met114, both of importance for anchoring ACh to the complementary side. Further, Trp53 in *Ls*-AChBP was found to possess an unexpected degree of flexibility. Functional studies on α4β2 nAChRs showed that mutations of the corresponding tryptophan affected ACh-mediated gating, underscoring the importance of the cation-π interaction. The work points to new aspects of the interactions between the ubiquitous neurotransmitter ACh and its receptors, which improves the foundation for rational drug design targeting nAChRs.

## Materials and Methods

### Materials

ACh (A9101) and all other chemicals were of analytical grade and purchased from Sigma-Aldrich (Sigma-Aldrich Denmark ApS) unless otherwise specified.

### Protein expression

*Ls*-AChBP (plasmid kindly provided by Dr. Titia K. Sixma) was expressed using the Bac-to-Bac baculovirus expression system in *Sf*9 insect cells. Purification was carried out by ion-exchange chromatography as previously described [15]. A solution of ∼2 mg/ml protein and 100 mM ACh (>200 times molar excess of ACh) in 0.1 M TRIS pH 8.0 and 0.2 M NaCl was equilibrated at 4°C for 1 hour before experiments.

### Crystallization

Crystals were obtained by the vapor diffusion method in sitting drops at 20°C. The drops consisted of 1 μl protein-ligand solution mixed with 1 μl crystallization solution containing 0.1 M TRIS pH 8.0, 2.05 M (NH_4_)_2_SO_4_ and 3% PEG400. Rod-shaped crystals were obtained within one month. The best crystals were obtained after streak seeding with crushed crystals into a new drop that was allowed to stand for three weeks. Drops containing crystals were supplemented with glycerol in crystallization solution to a final glycerol concentration of 25% before the crystals were mounted in cryo-loops and flash-cooled in liquid nitrogen.

### X-ray data collection

Data were collected at MAX-lab in Lund, Sweden on beamline I911–3 at 101 K using a wavelength of 1.000 Å. Images were collected with oscillation angle of 0.2° on a MarMosaic 225 detector. The data were processed with XDS [29] and scaled using SCALA in CCP4i [30]). An R(free) set was created in SCALA from 5% of the total data.

### Structure determination

The structure was solved by molecular replacement using the PHENIX program suite [31]. The structure of *Ls*-AChBP in complex with 3-(dimethylamino)butyl dimethylcarbamate (DMABC) was used as search model (PDB entry 3ZDG, [23]).

Refinements were performed with PHENIX and the structure manually adjusted in COOT [32] until no significant improvements in statistics were observed. Non-crystallographic symmetry (NCS) restraints were used in the refinement steps. B factors were refined as individual isotropic values. The structure was validated using COOT and PHENIX. Figures were prepared using PyMOL (The PyMOL Molecular Graphics System. Version 1.5.0.1, Schrödinger, LLC).

### Radioactive ligand binding

The binding affinity of ACh was determined to a construct of *Ls*-AChBP linked to a 5HT3 receptor ion channel [17]. The *Ls*-AChBP-5HT3 receptor chimera was stably expressed in HEK-293 cells (CRL-1573, American Type Culture Collection, Manassas, VA). Cell homogenate was prepared for binding experiments as previously described [33]. 800 μl homogenate suspension was mixed with 100 μl ACh solution in 48% EtOH and 100 μl of a ∼0.3 nM [^3^H]-epibatidine solution in 48% EtOH. The specific concentration of the epibatidine solution was determined by liquid scintillation counting. Binding was terminated after incubation for 1 hour by filtration over GF/C glassfiber filters (Brandel Inc, Gaithersburg, USA) preincubated for 30 min with 0.1% polyethyleneimine. Nonspecific binding was determined by incubation with an excess of (-)-nicotine (30 μM). Filters containing protein and bound [^3^H]-epibatidine were individually incubated for at least 4 hours with 3 ml Ultima Gold (PerkinElmer, Waltham, USA). Radioactivity was then measured by liquid scintillation counting on a Tri-Carb counter (PerkinElmer, Waltham, USA) and IC_50_ values were determined by nonlinear fit using GraphPad Prism 4, with all points in one experiment determined in triplicate. The K_i_ value was determined from the average IC_50_ of three individual experiments by the Cheng-Prusoff equation: K_i_ = IC_50_/(1+L/K_d_), where L is the concentration of [^3^H]-epibatidine used in the assay and K_d_ is the affinity of [^3^H]-epibatidine for the binding site (0.097 nM).

### Electrophysiology

Mutations were introduced by site directed mutagenesis as previously described [22] into plasmid expression vectors coding for human α4 and β2 nAChR subunits [34], [35], and confirmed by sequencing (Eurofins MWG Operon, Germany). Custom designed oligos were ordered from Eurofins MWG Operon (Germany). cRNA was produced using the mMessage mMachine T7 Transcription kit (Invitrogen Life Technologies Europe BV, Denmark) following the manufacturers protocol.

*Xenopus laevi*s oocytes were prepared as previously described [17], [34], [35] and injected with approximately 25 ng of a 10∶1 mixture of RNAs coding for the nAChR α4 and β2 subunits, respectively. Following injection, eggs were incubated for 2-7 days at 18 °C in modified Barth's solution (90 mM NaCl, 1.0 mM KCl, 0.66 mM NaNO_3_, 2.4 mM NaHCO_3_, 10 mM HEPES, 2.5 mM sodium pyruvate, 0.74 mM CaCl_2_, 0.82 mM MgCl_2_, 100 μg/ml Gentamycin and pH adjusted to 7.5). For two-electrode voltage-clamp recordings oocytes were placed in a custom designed recording chamber and voltage clamped at a holding potential ranging from −40 to −80 mV using an Axon Geneclamp 500 B amplifier (Molecular Devices, LLC, USA). Pipette resistances were 0.6–2.0 MΩ in OR2 (90 mM NaCl, 2.5 mM KCl, 2.5 mM CaCl_2_, 1.0 mM MgCl_2_, 5.0 mM HEPES and pH adjusted to 7.5). Fresh solutions of ACh in OR2 were prepared on the day of the experiment and applied to the oocytes with a flow rate of 2.0 ml/min via a glass capillary. Signals were low-pass filtered at 20 Hz and digitized at 200 Hz by an Axon Digidata 1322A (Axon, UK). Traces were recorded in Clampex 9.2 and subsequently analyzed using Clampfit. All data sets were constructed from at least five oocytes and conducted at minimally two individual days.

For data analysis, traces were baseline subtracted and responses to individual applications were read as peak current amplitudes. Curves were each constructed from two sets of data points in factor 10 concentration steps, *i.e.* for the red curve (Figure 4B) one set started at 0.0316 μM of ACh and the other at 0.1 μM. Each set was fitted in GraphPad Prism 4 and normalized to the maximum fitted value before the sets were combined. Concentration-response relationships were fitted by nonlinear regression to monophasic and biphasic concentration-response relationships and the best fit was determined by an F test in GraphPad Prism 4. Hill slopes were constrained to 1 and the starting point for curves set to zero. Responses were then normalized to the maximum fitted value and experiments from different eggs were combined.

### Accession number

The atomic coordinates and structure factors for the *Ls*-AChBP crystal structure with ACh has been deposited in the Protein Data Bank under accession number 3WIP.

## Supporting Information

Figure S1 **The** ***Ls*** **-AChBP binding pockets accommodate ACh tightly, especially on the principal side of the interface.** (**A**) Surface representation of the principal side of the interface viewed from the location of the complementary side. The area colored lime-green corresponds to residues within 5 Å of ACh. (**B**) Surface representation of the complementary side of the interface viewed from the principal side. The area colored yellow corresponds to residues within 5 Å of ACh.
(TIF)

Figure S2 **2mFo-DFc omit map (grey mesh) contoured at 1σ and carved around ACh is shown along with a positive difference density mFo-DFc map and a negative difference density mFo-DFc map (red mesh), both contoured at 3σ.** ACh is shown in sticks representation. Residues within 5 Å of ACh are shown in line representation (carbon green on principal side, carbon orange on complementary side, nitrogen blue, oxygen red and sulphur yellow). One water molecule is shown as red sphere. The hydrogen-bonding network through the water molecule is shown as black dashed lines.
(TIF)

Figure S3 **Location and electron density of one out of four PEG400 molecules found near an ACh binding pocket in the reported structure.** ACh (green) bound to *Ls*-AChBP, as reported here, is shown with the principal side of the interface in green cartoon representation and the complementary side in orange representation, with Trp53 shown in stick representation. A nearby PEG400 molecule is also shown in stick representation along with a 2mFo-DFc omit map calculated in PHENIX, displayed at 1σ and carved at 2 Å around PEG400. On top of this, the structure of ACh bound to a MMTS-modified Tyr53Cys mutant of *Ac*-AChBP (20) was superimposed in PyMOL. The two ACh molecules in the binding pocket are shown as cyan sticks. Different conformations of ACh in the *Ls*-AChBP and *Ac*-AChBP structures are observed. The additional ACh molecule in the *Ac*-AChBP structure is only possible because of the absence of Trp53 seen in *Ls*-AChBP.
(TIF)
