# Supplementary figures and images for: Molecular Recognition of the Neurotransmitter Acetylcholine by an Acetylcholine Binding Protein Reveals Determinants of Binding to Nicotinic Acetylcholine Receptors
Source: PLoS One. 2014 Mar 17;9(3):e91232. doi: 10.1371/journal.pone.0091232 (PMC3956608; doi:10.1371/journal.pone.0091232)

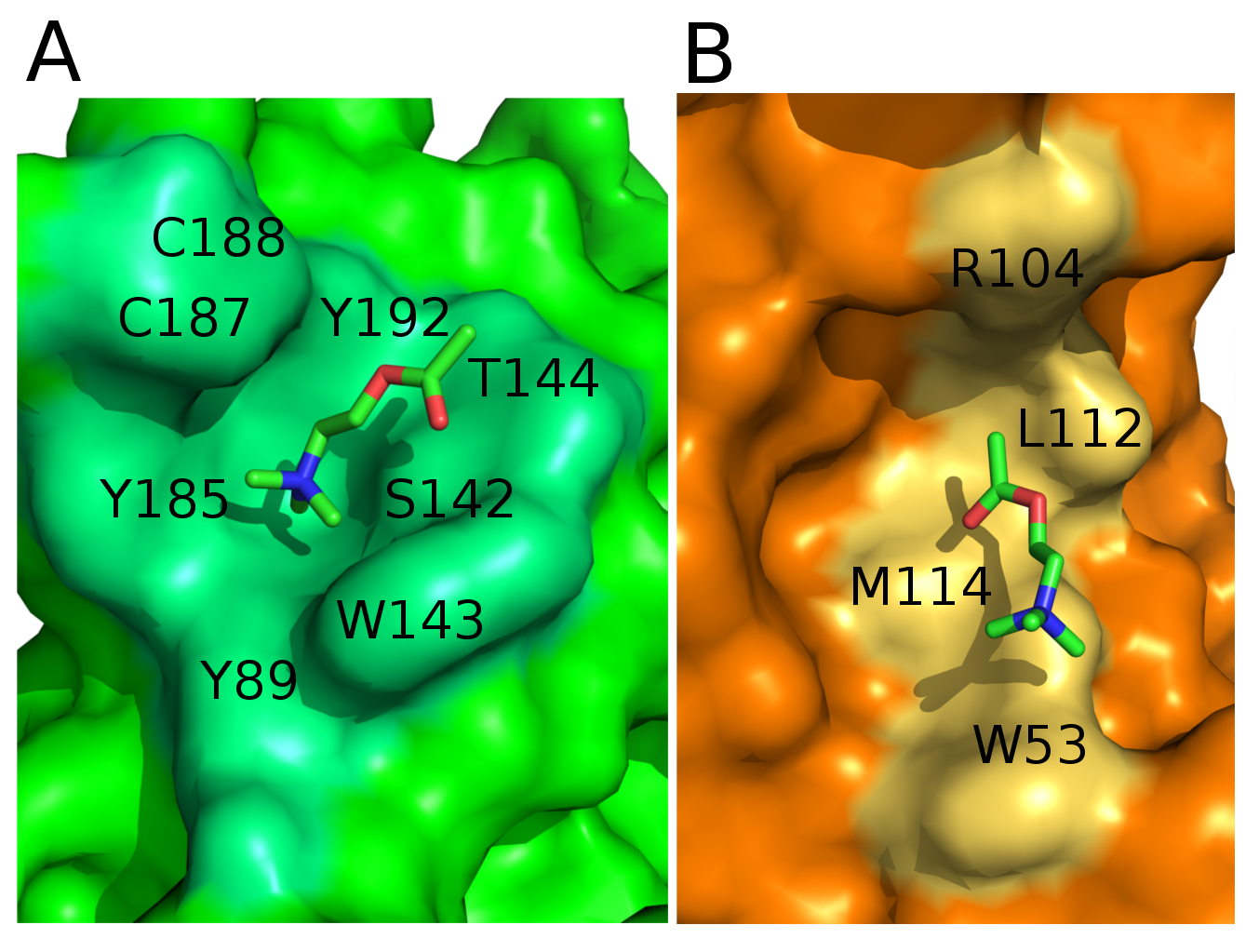

Supplement: Figure S1 — The Ls -AChBP binding pockets accommodate ACh tightly, especially on the principal side of the interface. (A) Surface representation of the principal side of the interface viewed from the location of the complementary side. The area colored lime-green corresponds to residues within 5 Å of ACh. (B) Surface representation of the complementary side of the interface viewed from the principal side. The area colored yellow corresponds to residues within 5 Å of ACh. (TIF) [file pone.0091232.s001.tif]

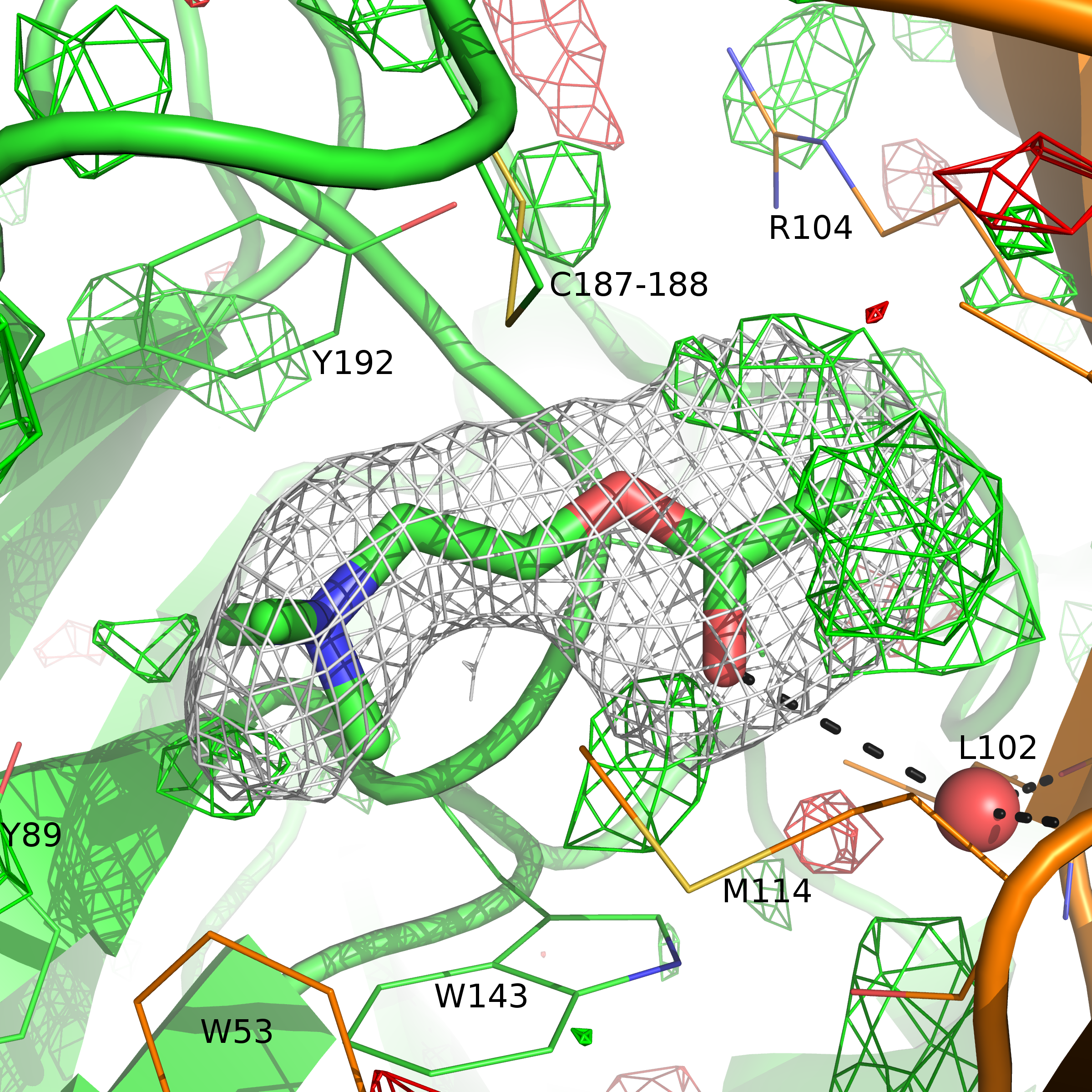

Supplement: Figure S2 — 2mFo-DFc omit map (grey mesh) contoured at 1σ and carved around ACh is shown along with a positive difference density mFo-DFc map and a negative difference density mFo-DFc map (red mesh), both contoured at 3σ. ACh is shown in sticks representation. Residues within 5 Å of ACh are shown in line representation (carbon green on principal side, carbon orange on complementary side, nitrogen blue, oxygen red and sulphur yellow). One water molecule is shown as red sphere. The hydrogen-bonding network through the water molecule is shown as black dashed lines. (TIF) [file pone.0091232.s002.tif]

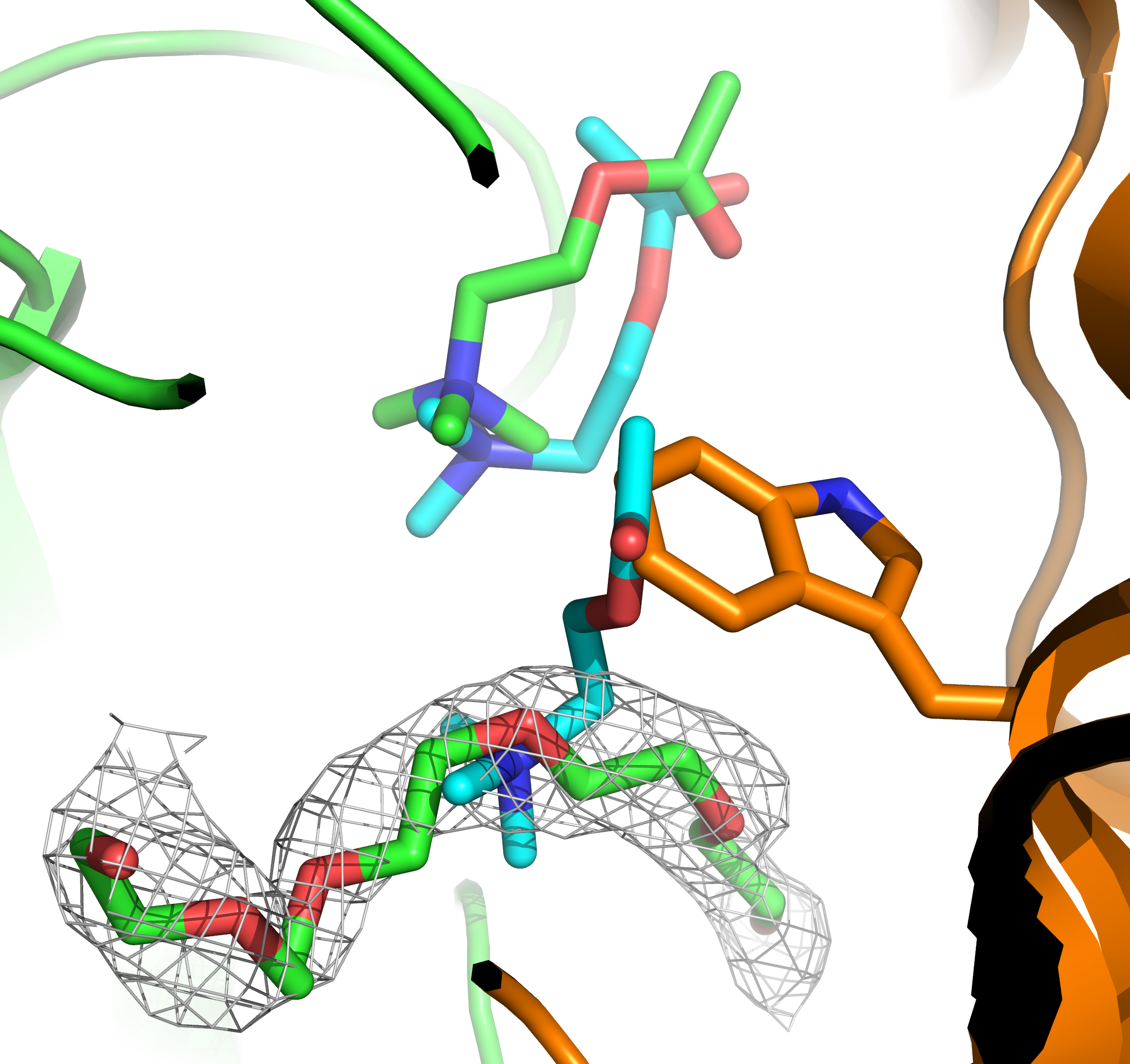

Supplement: Figure S3 — Location and electron density of one out of four PEG400 molecules found near an ACh binding pocket in the reported structure. ACh (green) bound to Ls-AChBP, as reported here, is shown with the principal side of the interface in green cartoon representation and the complementary side in orange representation, with Trp53 shown in stick representation. A nearby PEG400 molecule is also shown in stick representation along with a 2mFo-DFc omit map calculated in PHENIX, displayed at 1σ and carved at 2 Å around PEG400. On top of this, the structure of ACh bound to a MMTS-modified Tyr53Cys mutant of Ac-AChBP (20) was superimposed in PyMOL. The two ACh molecules in the binding pocket are shown as cyan sticks. Different conformations of ACh in the Ls-AChBP and Ac-AChBP structures are observed. The additional ACh molecule in the Ac-AChBP structure is only possible because of the absence of Trp53 seen in Ls-AChBP. (TIF) [file pone.0091232.s003.tif]
